# The Mouth as a Pocket

**Published:** 1881-01

**Authors:** 


### THE MOUTH AS A POCKET.

PUTTING coins, pins, needles and sundry other articles in the mouth for temporary safe-keeping is a practice that cannot be too strongly condemned. Coins circulate among all classes of people, and are frequently used for purposes not the most cleanly. It is highly probable that diseases have been communicated in this way. Very young children are inclined to put things in their mouths; and if the habit is not promptly checked, it often results in serious accidents, as the swallowing of coins and other articles. It is well to remember that silver coins are not poisonous, and that copper coins are, and that fatal results frequently follow when they get into the stomach. When, therefore, this happens, oil, lard, butter, or the whites of eggs should be given, which will form a coating around the coin, and prevent its being acted upon by the acids of the stomach. The diet should be of milk, eggs, rice or bread. A carthartic of castor oil should be given, and no acids taken into the stomach until the coin is passed out of the system.

Should the acids of the stomach be permitted to act on the coin, sufficient poison is soon formed to destroy life. Such a death is terrible, being brought about by unceasing retching and vomiting. The lives of many persons are lost through swallowing pins, needles and similar substances that were being held in the mouth.

---

WHAT IS LIBERTY?—There can be no absolute freedom. Every man is limited by those about him. You are free to be a good citizen. You are not free to murder, to steal, or to defraud. If you do these things, you will be put in confinement. You are free to be a kindly, courteous and cultivated member of society. You are not free to be morose, indecent and low-bred. If you are of this latter sort, people avoid you. You are free to speak wise and pure thoughts. You are not free to advocate folly and wickedness. You are free to be a law-abiding and peaceful member of the community; you are not free to be a Mormon or a cannibal You are free to utter those ideas which help man; you are not free to utter those ideas which destroy morality and corrupt the character of people. You are, in short, free to do right, but not to do wrong; free to speak the truth, but not to lie.

WITHERED leaves having the yellow, brown or red autumnal colors can be made green again by steeping them in water with a little zinc powder.

